# Arsenic exposure and the seroprevalence of total hepatitis A antibodies in the US population: NHANES, 2003–2012

**DOI:** 10.1017/S0950268815003088

**Published:** 2016-01-07

**Authors:** A. CARDENAS, E. SMIT, J. W. BETHEL, E. A. HOUSEMAN, M. L. KILE

**Affiliations:** School of Biological and Population Health Sciences, College of Public Health and Human Sciences, Oregon State University, Corvallis, OR, USA

**Keywords:** Antibody responses, arsenic, hepatitis A, hepatitis immunization, immunotoxicity, NHANES

## Abstract

We evaluated the association between urinary arsenic and the seroprevalence of total hepatitis A antibodies (total anti-HAV: IgG and IgM) in 11 092 participants aged ⩾6 years using information collected in the US National Health and Nutrition Examination Survey (2003–2012). Multivariate logistic regression models evaluated associations between total anti-HAV and total urinary arsenic defined as the sum of arsenite, arsenate, monomethylarsonate and dimethylarsinate (TUA1). Effect modification by self-reported HAV immunization status was evaluated. Total anti-HAV seroprevalence was 35·1% [95% confidence interval (CI) 33·3–36·9]. Seropositive status was associated with higher arsenic levels and this association was modified by immunization status (*P* = 0·03). For participants that received ⩾2 vaccine doses or did not know if they had received any doses, a positive dose-response association was observed between increasing TUA1 and odds of total anti-HAV [odds ratio (OR) 1·42, 95% CI 1·11–1·81; and OR 1·75, 95% CI 1·22–2·52], respectively. A positive but not statistically significant association was observed in those who received <2 doses (OR 1·46, 95% CI 0·83–2·59) or no dose (OR 1·12, 95% CI 0·98–1·30). Our analysis indicates that prevalent arsenic exposure was associated with positive total anti-HAV seroprevalence. Further studies are needed to determine if arsenic increases the risk for incident hepatitis A infection or HAV seroconversion.

## INTRODUCTION

Hepatitis A virus (HAV) is transmitted via the faecal–oral route and infection causes self-limiting liver disease which is generally asymptomatic or mild but can presents with more severe symptoms including fever, jaundice and occasionally fulminant hepatitis. Globally, there are about 1·4 million cases of acute hepatitis A per year with seroprevalence data suggesting that HAV affects tens of millions of people annually. In parts of the world with poor sanitation infrastructure, HAV infections tend to occur early in life with an estimated 90% of children having an infection before the age of 10 years. However, in developed countries with improved sanitation this pattern shifts and HAV infections tend to occur later in life in adolescents, adults and high-risk groups [1]. In the United States, there were 1398 reported cases of acute HAV in 2011 with the actual number of cases estimated to be between 1650 and 4370 [2]. Of these reported cases, 78% indicated they had not participated in any risk behaviours for HAV transmission such as travel to an endemic area, men having sex with men (MSM), injection drug use, or sex with multiple partners; nor had they been exposed to any known infected individuals [2].

In 1995, a vaccine against HAV became available in the United States which markedly reduced HAV incidence particularly after it was recommended as a routine vaccination for children in 1999. The vaccine was introduced incrementally with a 1996 recommendation from the Advisory Committee on Immunization Practices to vaccinate children living in communities with the highest disease rates followed by states and counties with elevated incidence rates [3]. Hepatitis A vaccine in children is administered in a two-dose series to children at age 12 months but it can also be provided during subsequent visits if the child has not been vaccinated at age 2 years. Adult immunization is recommended for international travellers to countries where hepatitis A is endemic, MSM, illegal drug users and other susceptible groups administered as a three-dose series along with the hepatitis B vaccine [4].

In 2009 hepatitis A immunization coverage estimates in the United States for ⩾1 and ⩾2 vaccine doses ranged from 29–58% and 6–24%, respectively [5]. Vaccination against HAV is very effective and studies show that protective levels of anti-HAV antibodies develop in 94–100% of adults 1 month after one dose of the vaccine and 100% have protective HAV antibody levels after two doses [6]. In children and adolescents, 97–100% have protective antibody levels 1 month after the first dose and 100% reach protective levels 1 month after the second dose [6]. Additionally, a follow-up study observed that after 5 years seroprotection rates were 99·7% and 100% in 5-year-old children who had received one and two doses, respectively [7].

Following inoculation, the immune system responds by producing immunoglobulin G (IgG) antibodies to the HAV. However, immunoglobulin M (IgM) antibodies have been detected 2–3 weeks after vaccination in 8–20% of adults. In the case of HAV infection, IgM anti-HAV appear after 5–10 days before the onset of symptoms but gradually disappear with increasing IgG levels conferring long-term protection against the virus. Subsequently, epidemiologists use total anti-HAV (IgM and IgG) levels to estimate the prevalence of previous hepatitis A infection [8]. Yet despite the availability of an effective vaccine, hepatitis A remains a public health problem particularly in highly endemic regions such as Central America, South America, Africa, Middle East, South East Asia and the South Pacific that have not fully implemented vaccination programmes [9, 10].

Previous studies indicate that the severity and morbidity of infectious diseases can be related to environmental pollutants and exogenous chemical exposures even at low levels [11]. The impact of environmental contaminants on the immune system has been documented in several epidemiological studies in the past decade. For example, an increased incidence of infections in children has been observed with elevated exposure to dioxins, polychlorinated biphenyls and polycyclic aromatic hydrocarbons [12, 13]. Emerging evidence also suggests that arsenic is an immunotoxicant [14]. Data from *in vitro* and *in vivo* studies show that arsenic influences macrophage differentiation and T-cell proliferation [15] and viral pathology [16]. Recent epidemiological studies report that elevated arsenic exposure during fetal development increases susceptibility to infectious diseases, including respiratory diseases, diarrhoea and overall mortality in children [17–20]. In addition, our group showed that urinary arsenic levels were inversely associated with varicella zoster virus IgG seroprevalence in the US population aged ⩾6 years [21]. Furthermore, a recent study in pregnant women reported that urinary arsenic levels was associated with increased odds of incident hepatitis E seroconversion suggesting that arsenic exposure during pregnancy may enhance susceptibility to hepatitis E viral infection [22].

While the most effective way to reduce the global burden of hepatitis A infection and transmission is through active immunization and improved sanitation, identifying environmental factors that can affect infectious diseases could inform novel prevention strategies to reduce the global burden of infectious diseases. Subsequently, the objective of this study was to investigate the cross-sectional association between arsenic exposure and immune response to HAV in a representative sample of the US population. Given that the National Health and Nutrition Examination Survey (NHANES) only measured total anti-HAV which cannot differentiate between antibodies produced by natural infection or vaccination, we limit our hypothesis to determining if there was a significant relationship between urinary arsenic levels and positive seroprevalence of total antibodies to HAV. Furthermore, we hypothesized that hepatitis A immunization would modify this association after adjusting for other risk factors because the production of antibodies (IgG) produced by vaccination be lower than levels produced after a natural infection.

## METHODS

### Study population

We used data from the NHANES, a representative cross-sectional survey designed to assess the health and nutritional status of the US population, from five consecutive cycles: 2003–2012. Response rates for the five NHANES consecutive cycles were 76%, 77·36%, 75·4%, 77·3% and 69·5% [23]. Serological markers for HAV were collected for participants aged ⩾2 years while urinary arsenic measurements were collected from participants aged ⩾6 years in all five survey cycles. Total hepatitis A antibodies were tested for all study participants while urinary arsenic was measured in one-third of a subsample for each survey cycle. Analytical methods for arsenic speciation and hepatitis A serology were consistent across survey cycles.

Individuals were included in this analysis if they were sampled for both urinary arsenic and total hepatitis A serology. Participants with a positive or equivocal HIV test were excluded from the analysis to avoid confounding by immunodeficiency (*n* = 20). Participants with an indeterminate serological test for HAV were also excluded from the analysis (*n* = 1). Analyses were further restricted by the availability of covariates. Total urinary arsenic was determined from urinary metabolites using two complementary approaches. Total urinary arsenic 1 (TUA1) defined as the sum of inorganic arsenic metabolites, and total urinary arsenic 2 (TUA2) defined as total urinary arsenic minus non-toxic arsenosugar metabolites described in detail in the Methods section. Depending on the exposure assessment approach, this resulted in 11 092 (TUA1) and 10 801 (TUA2) participants in the final analyses. Informed consent was obtained from all survey participants and study protocols were approved by the National Center for Health Statistics (NCHS) Research Ethics Review Board [24].

### Serological testing

At the time of examination participants provided blood via venepuncture. The samples were processed by the Division of Viral Hepatitis, CDC using a commercial assay (Vitros, Ortho-Clinical Diagnostics, USA) and determined total antibody (IgG and IgM) to the HAV (total anti-HAV). Results are obtained as a normalized signal, relative to a lot-specific calibration cut-off value [signal/cut-off (s/c)]. The total anti-HAV test relies on a photoluminescent reaction that is present for negative samples. Subsequently ratios <1 are considered a positive result. NHANES evaluates the anti-HAV results as antibody positive (<0·80 s/c), borderline (⩾0·80 and <1·0 s/c), antibody negative (⩾1·0 and <4·0 s/c) or retest (⩾4·0 s/c) [25]. Thus, being positive for total anti-HAV (seropositive) indicates the person has been previously infected with HAV or has been vaccinated against HAV. Being negative for anti-HAV (seronegative) indicates the person has not been infected with HAV or has not been vaccinated against HAV. NHANES only reports results as positive, negative or intermediate as quantitative results cannot be correlated to an endpoint titre [25].

### Self-reported immunization

Participants were asked to recall their hepatitis A immunization history for all NAHNES cycles and subsequently classified as receiving: ⩾2 doses, <2 doses, no dose, refused, and ‘don't know’. Participants that refused to answer the question were excluded from the analysis (*n* = 7).

### Urinary arsenic assessment

The analytical method to quantify arsenic metabolites in urine has been previously described [26] and consistent across cycles although the sensitivity of the reporting improved in 2011. Briefly, spot urine samples were obtained at the time of the physical examination in arsenic-free containers, shipped on dry ice, stored at ⩽–70 °C and analysed within 3 weeks of sampling at the Environmental Health Sciences Laboratory using high-performance liquid chromatography coupled to an inductively coupled-plasma dynamic reaction cell-mass spectrometry [25]. The limits of detections (LOD) for the 2003–2010 survey cycles were as follow: arsenate (1·0 *µ*g/l), arsenite (1·20 *µ*g/l), dimethylarsonic acid (1·70 *µ*g/l), monomethylarsonic acid (0·90 *µ*g/l), arsenobetaine (0·40 *µ*g/l), arsenocholine (0·60 *µ*g/l) and TUA (0·74 *µ*g/l). The limits of detection for the 2011–2012 survey cycle were slightly different: arsenate (0·87 *µ*g/l), arsenite (0·48 *µ*g/l), dimethylarsonic acid (1·80 *µ*g/l), monomethylarsonic acid (0·89 *µ*g/l), arsenobetaine (1·19 *µ*g/l), arsenocholine (0·28 *µ*g/l) and TUA (1·25 *µ*g/l). The proportion of participants that were below the LOD for the combined survey cycles was 95·9% for arsenate, 90·1% for arsenite, 67·6% for monomethylarsonic acid, 18·0% for dimethylarsonic acid, 40·86% for arsenobetaine, 97·60% for arsenocholine, and 1·28% for TUA.

If a metabolite was below the LOD, a value equivalent to the LOD divided by the square root of 2 was assigned. TUA1 was defined as the sum of arsenate, arsenite, dimethylarsonic acid, and monomethylarsonic acid. Additionally, we defined TUA2 as the total urinary arsenic minus arsenobetaine and arsenocholine as a complementary approach to evaluate total arsenic exposure. For TUA2, 211 participants had urinary arsenobetaine and arsenocholine levels greater than TUA, yielding negative levels for TUA2. This would indicate that these individuals had recently consumed seafood which is naturally high in these compounds [27]. Since these organoarsenic compounds are considered to be less toxic than inorganic arsenic and its metabolites, these participants were excluded from analyses that used TUA2. In sensitivity analyses replacing these 211 values with the LOD for TUA divided by the square root of 2 did not affect the observed association (results not shown). Both approaches used to model total urinary arsenic (TUA1 and TUA2) concentration should not be influenced by organoarsenic compounds found in seafood [28].

### Covariates

Previous research has described differences in urinary arsenic levels by age, sex, race and gender in the US population [28]. Additionally, the prevalence of HAV has been shown to differ by age, gender, race and country of birth [29]. Subsequently, these covariates were extracted from the five NHANES cycles and further evaluated for adjustment in multivariate models. Race/ethnicity was reported as non-Hispanic white, non-Hispanic black, Mexican American, other Hispanic and other race including multiracial. Economic status was evaluated by using the family's income to poverty ratio. Body mass index (BMI) was reported in kg/m^2^. As suggested by the NCHS, the effect of spot urine dilution was accounted for by adjusting for creatinine (mg/dl) concentration in all multivariate models. Survey year was included in the analysis to control for temporal changes in HAV serology and arsenic exposure.

### Statistical analysis

Sampling weights were calculated for the arsenic subsample of NHANES from 2003 to2012 by rescaling the survey weights of each cycle by multiplying the arsenic subsample weight by 1/5, as recommended by the analytical guidelines of NHANES [30]. To account for the complex survey design implemented by NHANES, all analyses used survey weights, primary sampling units and strata. However, we also conducted a sensitivity analysis by removing the weights. Statistical significance was evaluated using a cut-off value of *α* ⩽ 0·05, and all tests performed were two-tailed. Standard errors (s.e.s) and 95% confidence intervals (CIs) were estimated using the Taylor linearization method. All statistical analyses were performed using Stata v. 12.1 (StataCorp LP, USA).

The distribution of TUA1, TUA2 and creatinine were right-skewed and subsequently natural log-transformed. The geometric mean and the s.e. for total arsenic (TUA1 and TUA2) was estimated across categories of sex, race, family income/poverty ratio, BMI, age, country of birth, and seroprevalence of total anti-HAV and evaluated using linear regression models and a Wald test adjusted for creatinine concentration to account for urine dilution. Bivariate associations between all covariates and total-anti HAV seroprevalence were evaluated and adjusted for in multivariate models if differences were observed. Multivariate logistic regression models were used to estimate the odds of being seropositive for total anti-HAV adjusting for log-transformed creatinine (continuous), age (continuous), sex, race, family income/poverty ratio (continuous), country of birth, BMI (continuous) and survey year. To evaluate effect modification the multiplicative interaction between log-transformed TUA1 and TUA2, and self-reported vaccination status were tested using a Wald test adjusting for all covariates. Odds ratios (ORs) and 95% CIs were estimated for each level of self-reported hepatitis A immunization from adjusted models that included the multiplicative interaction between exposure and immunization history to estimate the association between total arsenic exposure and hepatitis A seroprevalence.

## RESULTS

The geometric means of TUA for selected population characteristics are presented in Table 1. Overall, TUA was slightly higher for females, in individuals reporting to be multiracial or of other race followed by Mexican Americans, and in individuals living in households above the poverty level for TUA2 but not for TUA1. BMI was also associated with TUA and with age. Individuals born in the United States had significantly lower TUA compared to individuals born elsewhere. Urinary arsenic was higher in seropositive anti-HAV participants compared to seronegative individuals. No differences in TUA were observed by self-reported immunization status to HAV.

**Table 1.** Weighted geometric mean (GM) and standard error (s.e.) of total urinary arsenic levels (*μ*g/l) adjusted for log-transformed creatinine, NHANES 2003–2012

	TUA1*		TUA2†	
Population characteristic	(*N* = 11092)	GM (s.e.)‡	(*N* = 10 801)	GM (s.e.)‡
Overall		6·34 (1·01)		5·06 (1·02)
Sex		*P* = 0·026		*P* = 0·074
Male	5517	6·40 (1·02)	5408	5·19 (1·02)
Female	5575	6·58 (1·01)	5393	5·40 (1·03)
Race		*P* < 0·001		*P* < 0·001
Mexican American	2267	6·91 (1·02)	2222	5·93 (1·03)
Other Hispanic	821	8·07 (1·02)	799	7·19 (1·04)
Non-Hispanic white	4593	6·18 (1·02)	4443	4·89 (1·03)
Non-Hispanic black	2637	6·08 (1·02)	2588	4·84 (1·04)
Other race/multiracial	774	9·79 (1·04)	749	10·38 (1·06)
Family income/poverty ratio		*P* = 0·2885		*P* = 0·010
Below poverty level ⩽1	2771	6·38 (1·02)	2703	4·95 (1·03)
Above poverty level >1	8321	6·50 (1·01)	8098	5·37 (1·03)
Body mass index (kg/m^2^)		*P* < 0·001		*P* < 0·001
Underweight	264	6·44 (1·04)	257	5·53 (1·08)
Normal weight	4342	6·73 (1·02)	4218	5·59 (1·03)
Overweight	3132	6·58 (1·02)	3053	5·38 (1·03)
Obese	3354	6·12 (1·01)	3273	4·89 (1·03)
Age (years)		*P* < 0·001		*P* < 0·001
6–11	1420	7·06 (1·02)	1377	6·00 (1·03)
12–19	2226	5·68 (1·02)	2177	4·00 (1·03)
⩾20	7446	6·56 (1·01)	7247	5·46 (1·03)
Country of birth		*P* < 0·001		*P* < 0·001
United States	8863	6·16 (1·01)	8619	4·87 (1·03)
Elsewhere	2229	8·75 (1·02)	2182	8·65 (1·04)
Hepatitis A immunization		*P* = 0·104		*P* = 0·051
⩾2 doses	2941	6·68 (1·02)	2854	5·65 (1·03)
<2 doses	273	6·52 (1·03)	268	5·46 (1·08)
0 dose	6603	6·41 (1·01)	6438	5·20 (1·03)
Don't know	1275	6·52 (1·02)	1241	5·18 (1·04)
Hepatitis A (anti-HAV)		*P* < 0·001		*P* < 0·001
Seropositive	5064	7·12 (1·02)	4934	6·19 (1·03)
Seronegative	6028	6·17 (1·01)	5867	4·88 (1·03)

* TUA1 = As^III^ + As^V^ + MMA + DMA.

† TUA2 = total urinary As – arsenocholine – arsenobetaine.

‡ Models adjusted for log-transformed creatinine.

The total anti-HAV seroprevalence (e.g. seropositive) for selected population characteristics are presented in Table 2. Across all 10 years, the overall seropositive prevalence was estimated to be 35·1% (95% CI 33·3–36·9). Seropositive prevalence of total anti-HAV was highest in Mexican Americans (75%), participants living below the poverty level (45%), children aged 6–11 years (44%), participants in the lowest urinary creatinine tertile (38%), individuals not born in the United States (77%) and in individuals that self-reported receiving ⩾2 doses of hepatitis A vaccine (52%). Total anti-HAV seropositive prevalence was elevated for participants in the highest quartile of arsenic exposure compared to the third, second and first quartiles for TUA1 (43%) or TUA2 (42%). In our restricted sample for TUA1, a total of 26·51% individuals reported receiving ⩾2 doses of hepatitis A vaccine, 2·46% reported receiving <2 doses, 59·53% participants reported receiving no dose and 11·49% participants reported not knowing whether they received a dose of the HAV vaccine. A similar self-reported HAV immunization distribution was observed for TUA2.

**Table 2.** Hepatitis A seroprevalence (total anti-HAV) by demographic characteristics: NHANES: 2003–2012

	Hepatitis A seroprevalence (total anti-HAV)			
	TUA1*		TUA2†	
Population characteristics	(*N* = 11 092)	Seropositive (95% CI)	(*N* = 10 801)	Seropositive (95% CI)
Overall prevalence	5064	35·1% (33·3–36·9)	4934	34·9% (33·1–36·7)
Sex		*P* = 0·926		*P* = 0·936
Male	5517	35·0% (32·7–37·4)	5408	34·9 (32·5–37·3)
Female	5575	35·1% (33·1–37·1)	5393	34·9 (32·9–36·9)
Race		*P* < 0·001		*P* < 0·001
Mexican American	2267	75·5% (72·9–78·1)	2222	75·4% (72·8–78·1)
Other Hispanic	821	63·2% (58·5–68·0)	799	63·1% (58·4–67·8)
Non-Hispanic white	4593	25·5% (23·9–27·2)	4443	25·2% (23·5–26·9)
Non-Hispanic black	2637	36·9% (34·3–39·6)	2588	36·9% (34·4–39·6)
Other race/multiracial	774	57·8% (52·5–62·9)	749	57·8% (52·6–62·9)
Family income/poverty ratio		*P* < 0·001		*P* < 0·001
Below poverty level ⩽1	2771	45·4% (31·9–34·9)	2703	45·3% (41·8–48·9)
Above poverty level >1	8321	33·1% (31·4–34·9)	8098	32·9% (31·2–34·7)
Body mass index (kg/m^2^)		*P* = 0·138		*P* = 0·168
Underweight	264	31·6% (24·2–39·1)	257	32·6% (24·9–40·3)
Normal weight	4342	35·8% (33·2–38·4)	4218	35·8% (33·2–38·5)
Overweight	3132	35·9% (33·6–38·2)	3053	35·6% (33·4–37·8)
Obese	3354	33·6% (31·3–35·9)	3273	33·3% (30·9–35·7)
Age (years)		*P* < 0·001		*P* < 0·001
6–11	1420	44·2% (39·7–49·3)	1377	44·8% (39·6–49·9)
12–19	2226	36·4% (32·3–40·4)	2177	35·9% (31·6–40·3)
⩾20	7446	33·9% (33·4–37·4)	7247	33·8% (32·0–35·6)
Creatinine (mg/dl) (tertiles)		*P* < 0·001		*P* < 0·001
(6–75)	3367	37·9% (35·5–40·3)	3175	37·8% (35·3–40·2)
(76–145)	3850	36·7% (34·2–39·1)	3804	36·5% (34·1–38·9)
(146–800)	3875	30·7% (28·3–33·1)	3822	30·6% (28·2–33·0)
Country of birth		*P* < 0·001		*P* < 0·001
United States	8863	27·9% (26·3–29·6)	8619	27·7% (26·1–29·4)
Elsewhere	2229	76·9% (73·7–80·0)	2182	76·6% (73·3–79·9)
TUA1 (*μ*g/l) (quartiles)		*P* < 0·001		
2·69–4·14	2470	32·9% (30·2–35·6)	–	–
4·15–5·89	2755	32·4% (29·3–34·8)	–	–
5·90–8·54	2848	31·9% (29·4–34·5)	–	–
8·55–628	3019	43·1% (40·1–46·0)	–	–
TUA2 (*μ*g/l) (quartiles)				*P* < 0·001
0–2·72	–	–	2460	31·6% (29·1–34·1)
2·73–5·3	–	–	2661	33·6% (30·9–36·3)
5·31–9·88	–	–	2824	32·9% (29·7–36·0)
9·89–718·9	–	–	2856	41·8% (38·9–44·8)
Hepatitis A immunization		*P* < 0·001		*P* < 0·001
⩾2 doses	2941	51·9% (48·8–55·0)	2854	52·0% (48·9–55·2)
<2 doses	273	41·6% (33·3–49·8)	268	41·9% (33·6–50·4)
0 dose	6603	29·1% (27·3–30·9)	6438	28·8% (26·9–30·6)
Don't know	1275	35·0% (31·1–38·9)	1241	35·3% (31·5–39·1)

CI, Confidence interval.

* TUA1 = As^III^ + As^V^ + MMA + DMA.

† TUA2 = total urinary As – arsenocholine – arsenobetaine.

**Fig. 1.**
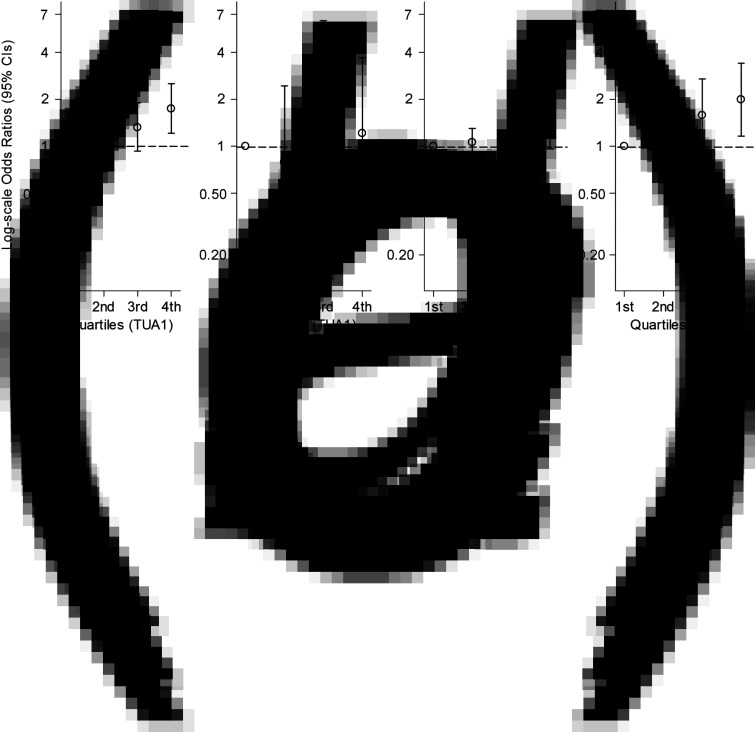
Adjusted odds ratios* for the association between total urinary arsenic (TUA1) in quartiles and total anti-HAV seropositive prevalence by self-reported HAV immunization: (*a*) received ⩾2 doses, (*b*) received <2 doses, (*c*) 0 dose and (*d*) ‘don't know’. [* Odds ratios from a logistic regression model that included the interaction between arsenic exposure by quartiles (TUA1) and hepatitis A immunization adjusted for log-transformed creatinine, age, sex, race, family income/poverty ratio, country of birth, body mass index and survey year.]

**Fig. 2.**
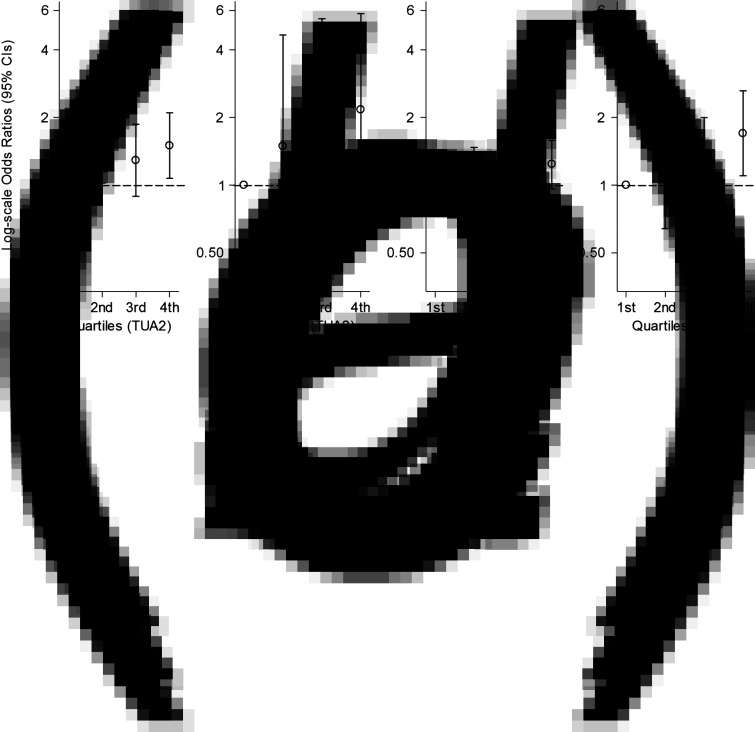
Adjusted odds ratios* for the association between total urinary arsenic (TUA2) in quartiles and total anti-HAV seropositive prevalence by self-reported HAV immunization: (*a*) received ⩾2 doses, (*b*) received <2 doses, (*c*) 0 dose and (*d*) ‘don't know’. [* Odds ratios from a logistic regression model that included the interaction between arsenic exposure by quartiles (TUA2) and hepatitis A immunization adjusted for log-transformed creatinine, age, sex, race, family income/poverty ratio, country of birth, body mass index and survey year.]

The ORs for the association between TUA and total anti-HAV seropositive prevalence were estimated for each self-reported category of hepatitis A immunization from adjusted logistic regression models that included the multiplicative interaction between log-transformed urinary arsenic (TUA1 and TUA2) and self-reported hepatitis A immunization (Table 3). In multivariate models the multiplicative interaction between continuous log-transformed TUA1 and self-reported hepatitis A vaccination as categorical outcome (⩾2 doses, <2 doses, no dose, ‘don't know’) was statistically significant (Wald *P* = 0·032) as well as with TUA2 (Wald *P* = 0·010) after adjusting for log-transformed creatinine, age, sex, race, BMI, family income/poverty ratio, country of birth and survey year. In adjusted analysis, participants that self-reported receiving the complete two doses of hepatitis A vaccine had 42% greater odds of testing positive for total anti-HAV for every unit increase in log-transformed TUA1 levels (OR 1·42, 95% CI 1·11–1·81). Similarly, the adjusted odds of testing positive for total anti-HAV (e.g. seropositive) increased by 17% for every unit increase in log-transformed TUA2 (OR 1·17, 95% CI 1·04–1·31).The odds of testing positive for total anti-HAV increased by 75% for every unit increase in log-transformed TUA1 levels in participants reporting not knowing whether they have received a hepatitis A vaccine dose (OR 1·75, 95% CI 1·22–2·52). A marginal increase of 20% was also observed for this group in the adjusted odds of being positive for total anti-HAV for every unit increase in log-transformed TUA2 (OR 1·20, 95% CI 0·97–1·48). No association was observed for participants that self-reported receiving <2 doses of hepatitis A vaccine for TUA1 (OR 1·46, 95% CI 0·83–2·59) or TUA2 (OR 1·26, 95% CI 0·95–1·67) or for individuals that reported receiving no dose of the vaccine for TUA1 (OR 1·12, 95% CI 0·98–1·30) or TUA2 (OR 1·07, 95% CI 0·99–1·16). Results were consistent when models were stratified by self-reported vaccination status or analysed without the sampling design and weights (results not shown). The observed relationship remained consistent when total arsenic was modelled by quartiles of exposure of TUA1 (Fig. 1) or TUA2 (Fig. 2). Age did not modify the observed association.

**Table 3.** Adjusted odds ratios* for the association of total urinary arsenic levels and total anti-HAV seropositive prevalence by self-reported immunization status

	⩾2 doses	<2 doses	0 dose	Don't know
Exposure assessment	aOR (95% CI)	aOR (95% CI)	aOR (95% CI)	aOR (95% CI)
Ln TUA1†	1·42 (1·11–1·81)	1·46 (0·83–2·59)	1·12 (0·98–1·30)	1·75 (1·22–2·52)
Ln TUA2‡	1·17 (1·04–1·31)	1·26 (0·95–1·67)	1·07 (0·99–1·16)	1·20 (0·97–1·48)

aOR, Adjusted odds ratio; CI, confidence interval.

* Odds ratios from a logistic regression model that included the interaction between arsenic exposure (TUA1 or TUA2) and hepatitis A vaccine adjusted for log-transformed creatinine, age, sex, race, family income/poverty ratio, country of birth, body mass index and survey year.

† TUA1 = As^III^ + As^V^ + MMA + DMA.

‡ TUA2 = total urinary As – arsenocholine – arsenobetaine (excluding negatives levels due to arsenocholine + arsenobetaine > total As).

## DISCUSSION

This cross-sectional study observed that higher arsenic exposure levels were associated with a greater probability of total anti-HAV seropositive prevalence in the US population aged ⩾6 years.

This dose-dependent association was consistently positive in all self-reported immunization strata but only reaches statistical significance in individuals that reported receiving ⩾2 doses of HAV immunization and for participants that did not know whether they had received HAV immunization. Given the cross-sectional nature of this study and reliance on total anti-HAV as the outcome, we cannot ascertain whether arsenic exposure is related to increased risk of hepatitis A infection or if toxic exposure modulates the immunological response to vaccination since a positive total anti-HAV test is not able to distinguish between a present, previous infection, or vaccine-induced immunity [31]. This is because hepatitis A IgM indicates a current underlying infection but hepatitis A IgG indicates immunity or recovery from a previous natural infection [32]. Furthermore, it is unknown if anti-HAV levels diminish over time. However, total anti-HAV is commonly used in epidemiological studies to measure the overall prevalence of previous infections [8]. The active production of the IgG antibody by vaccination can be 10- to 100-fold lower compared to IgG antibody levels produced by natural infections and in many instances present below the detection limit for many commercial assays including the assay used by NHANES [8]. This is also highlighted in our data that shows that only 52% of individuals that self-reported receiving ⩾2 doses of HAV vaccine tested seropositive for total anti-HAV. It is possible, however, that this could also originate from recall bias from participants as the sensitivity of self-reported HAV immunization has been estimated to be 63% compared to electronic medical records [33]. While we cannot rule out misclassification it is likely to be non-differential in relationship to urinary arsenic concentrations which is unlikely to be known by an individual. Thus, a possible explanation of our findings is that arsenic exposure may be associated with a lower protective immunity provided by the HAV immunization, which may subsequently result in HAV infection yielding a stronger antibody response.

This interpretation contradicted our initial hypothesis that HAV vaccination would be protective of the immunotoxic effect from arsenic exposure. Additionally, the absence of an association for individuals receiving no dose of the vaccine or <2 doses could be attributed to the lack of protection of not receiving the adequate immunization so differential exposure to arsenic, either high or low, would not affect the susceptibility to infection or immune response of these subgroups. The observed significant association for protected individuals (self-reported receiving two doses) or the mixture of protected/unprotected individuals (self-reporting not knowing whether they received any dose) could be influenced by arsenic exposure levels by either: (*a*) decreasing protecting immunity of the vaccine or (*b*) altering antibody response to the hepatitis A vaccine. Arsenic could contribute to the dysregulation of IgG or IgM antibody production in vaccinated participants yielding higher detectable levels of total anti-HAV. Additional studies are needed to test the hypothesis that arsenic reduces vaccine-induced immunity conferred by HAV vaccination or whether it actually increases susceptibility to hepatitis A infection. A recent epidemiological investigation demonstrated that elevated urinary arsenic levels during pregnancy increase the incidence of hepatitis E virus seroconversion, suggesting that environmental arsenic exposure enhances susceptibility to hepatitis E viral infection [22]. Further studies are therefore warranted to determine if arsenic exposure can influence susceptibility to hepatitis A.

There is considerable evidence that arsenic affects the immune system. Experimental studies in zebrafish have demonstrated that exposure of arsenic at environmentally relevant concentrations compromised the overall innate and adaptive immune system [34, 35]. *In vivo* studies have also shown that arsenic exposure increases the percentage and total levels of CD8+ T cells, and decreases cytokine production [36]. Alterations in gene expression related to the immune response, including genes involved in T-cell receptor signalling have also been observed with increased exposure to inorganic arsenic [37, 38]. T cells play a key role in the immune response to infections and vaccine-induced immunity that could modulate arsenic's induced immunotoxicity [39]. Recent evidence from birth cohorts have demonstrated that *in utero* exposure to arsenic altered the CD4 + /CD8 + T-cell ratios and also slightly decreased the proportion of B cells in newborns [40, 41]. The key role of T cells in stimulating B cells for the production of antibodies makes T-cell regulation a likely target for the observed association.

This study has several strengths including the use of a large recently collected representative sample of the US population across a time period of 9 years. Arsenic exposure levels represent exposure at environmentally relevant concentrations and our complementary approach of using two different methods for assessing exposure yielded consistent results. The use of several biomarkers of arsenic exposure and the meticulous quality control procedures for the analysis and collection of specimens implemented by NHANES are also important strengths. However, our study also has several limitations. Primarily, total anti-HAV seropositivity is unable to differentiate between a present or previous infection and vaccine-induced immunity. Furthermore, the assay used by NHANES is qualitative and cannot be correlated to a titre endpoint. The data were collected cross-sectionally and therefore we cannot evaluate temporality of this association between arsenic and anti-HAV response. While we were able to adjust for major risk factors of HAV infection identified in the literature, the possibility of residual confounding cannot be ruled out. Moreover, despite the strengths of using a biomarker of internal dose to ascertain arsenic exposure, urinary arsenic was measured only at one point in time and we acknowledge that exposure may fluctuate over time. The possibility for reverse causation of viral hepatitis modifying the metabolism and excretion of urinary arsenic cannot be ruled out and should be considered in future studies. NHANES does not sample institutionalized or homeless individuals and it is expected that the prevalence of HAV would be much higher in these individuals.

## CONCLUSION

Overall, elevated arsenic exposure was positively associated with the seroprevalence of total anti-HAV in participants receiving ⩾2 doses of HAV vaccine and for individuals unable to recall their immunization history after adjusting for major risk factors and potential confounders. The elevated prevalence of serological markers suggests that arsenic exposure may alter the immune response to HAV and this relationship was modified by self-reported immunization status. This study generates hypotheses for further study that are designed to understand whether arsenic exposure alters the immune response to hepatitis A or alters the response to vaccine-induced immunity.
